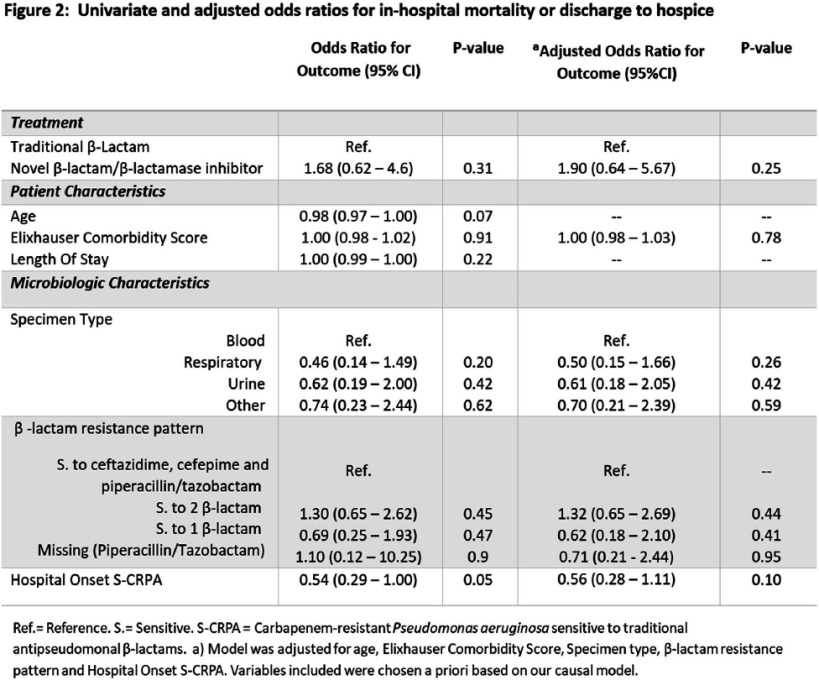# Mortality Risk Factors in Cases of Carbapenem-Resistant Pseudomonas aeruginosa Susceptible to Traditional Antipseudomonal β-lactams

**DOI:** 10.1017/ash.2025.358

**Published:** 2025-09-24

**Authors:** Elizabeth Kim, Jesse Jacob, Jessica Howard-Anderson

**Affiliations:** 1 Emory University School of Medicine; 2 Emory University

## Abstract

**Background:** Carbapenem-resistant Pseudomonas aeruginosa (CRPA) can cause healthcare-associated infections associated with poor outcomes. Unlike other carbapenem-resistant organisms, CRPA is often susceptible to at least one traditional anti-pseudomonal β-lactam antibiotic including cefepime, ceftazidime, and piperacillin-tazobactam (“S-CRPA”). This study aimed to determine if treatment of S-CRPA infections with a novel β-lactam/β-lactamase inhibitor (βL/ βLI) as opposed to a traditional antipseudomonal β-lactam which may increase the likelihood of resistance, was associated with improved clinical outcomes. **Methods:** We retrospectively analyzed all incident S-CRPA isolates in a four-hospital academic healthcare system in Atlanta, GA from 1/1/2013 to 9/30/2022. Patients receiving either a βL/ βLI or a traditional antipseudomonal β-lactam for definitive antibiotic therapy, defined as having received at least 3 days of the specified antibiotic between 4-14 days after the culture was obtained, were included. We excluded patients with cystic fibrosis. We compared patients who received definitive treatment with a βL/ βLI to those who received definitive therapy with a traditional anti-pseudomonal β-lactam. The primary outcome was mortality (in-hospital mortality and those discharged to hospice). We performed univariable and multivariable logistic regression analysis using SAS 9.4. A causal diagram was created to a priori to identify potential confounders for inclusion in a multivariate analysis including Elixhauser comorbidity score, specimen source, β-lactam resistance pattern, and whether the patient had a hospital-onset infection (culture obtained ≥3 days after admission) **Results:** There were 258 patients with S-CRPA who received definitive treatment with either βL/ βLI (n=22) or traditional anti-pseudomonal β-lactams (n= 236). Those who received definitive βL/ βLI therapy had higher Elixhauser comorbidity scores, longer lengths of stay, were more likely to have bacteremia, and more likely to have resistance to one or more traditional anti-pseudomonal β-lactams than those treated with traditional anti-pseudomonal β-lactams (Figure 1). In the univariable analysis, patients who received definitive BL/BLI therapy had increased mortality and patients without bacteremia and hospital-onset infection had decreased mortality, but the associations were not statistically significant (Figure 2). In the multivariable analysis, treatment with traditional anti-pseudomonal β-lactams was not significantly associated with mortality (OR 1.9 95%CI 0.6 – 5.7). **Conclusions:** In patients with S-CRPA we did not observe a significant difference in mortality comparing definitive antibiotic treatments. A low number of S-CRPA isolates treated with a βL/ βLI limited our ability to assess the true effect of traditional anti-pseudomonal β-lactams versus βL/ βLI on mortality.

**Figure:**
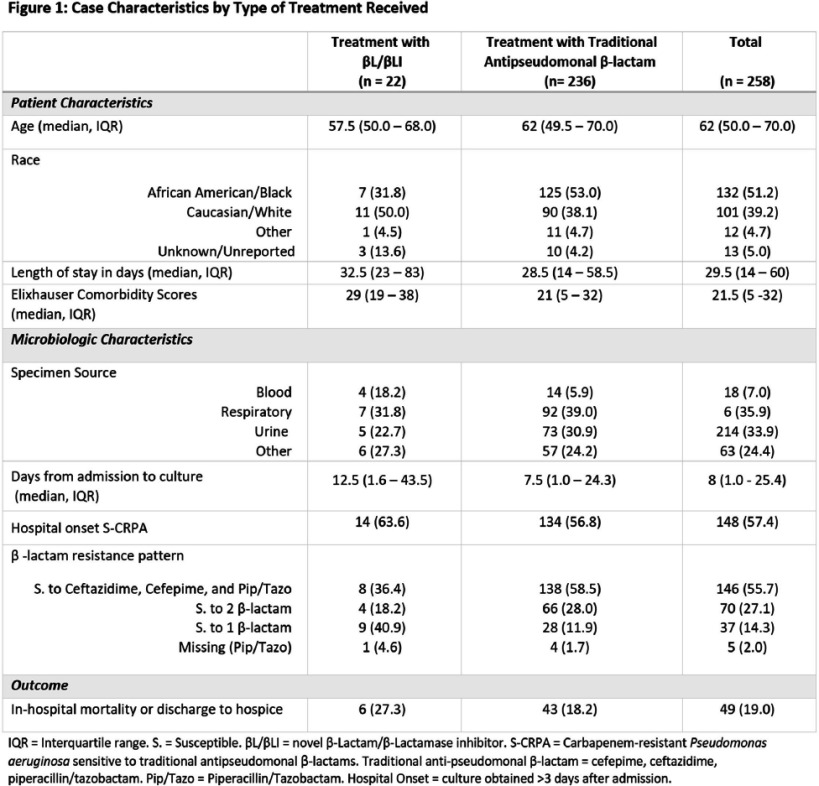

**Figure:**